# Between Air and Artery: A History of Cardiopulmonary Bypass and the Rise of Modern Cardiac Surgery

**DOI:** 10.3390/jcdd12090365

**Published:** 2025-09-18

**Authors:** Vasileios Leivaditis, Andreas Maniatopoulos, Francesk Mulita, Paraskevi Katsakiori, Nikolaos G. Baikoussis, Sofoklis Mitsos, Elias Liolis, Vasiliki Garantzioti, Konstantinos Tasios, Panagiotis Leventis, Nikolaos Kornaros, Andreas Antzoulas, Dimitrios Litsas, Levan Tchabashvili, Konstantinos Nikolakopoulos, Manfred Dahm

**Affiliations:** 1 Department of Cardiothoracic and Vascular Surgery, Westpfalz Klinikum, 67655 Kaiserslautern, Germany; vnleivaditis@gmail.com (V.L.); mdahm@westpfalz-klinikum.de (M.D.); 2 Department of Electrical and Computer Engineering, Democritus University of Thrace, 67100 Xanthi, Greece; amaniatopoulos@gmail.com; 3 Department of General Surgery, General Hospital of Eastern Achaia–Unit of Aigio, 25100 Aigio, Greece; vkatsak@gmail.com (P.K.); levedis6@gmail.com (P.L.); kornaros.nikolaos@hotmail.com (N.K.); tchabashvili.alexander@gmail.com (L.T.); 4 Department of Cardiac Surgery, Ippokrateio General Hospital of Athens, 11527 Athens, Greece; nikolaos.baikoussis@gmail.com; 5 Department of Thoracic Surgery, School of Medicine, Attikon University Hospital, National and Kapodistrian University of Athens, 12462 Athens, Greece; sophocmit@yahoo.gr; 6 Department of Oncology, General University Hospital of Patras, 26504 Patras, Greece; lioliselias@yahoo.gr; 7 Department of Surgery, General University Hospital of Patras, 26504 Patras, Greece; vigarant@yahoo.com (V.G.); kostastasiosmd@gmail.com (K.T.); a.antzoulas@hotmail.com (A.A.); 8 John Radcliffe Hospital Emergency Department, University Hospitals NHS Foundation Trust, Headley Way, Headington, Oxford OX3 9DU, UK; 9 Department of Surgery, General Hospital of Lamia, 35100 Lamia, Greece; dimlitsas@icloud.com; 10 Department of Vascular Surgery, General University Hospital of Patras, 26505 Patras, Greece; konstantinosn@yahoo.com

**Keywords:** cardiopulmonary bypass, heart–lung machine, extracorporeal circulation, medical history, ECMO, perfusion technology, surgical innovation

## Abstract

Cardiopulmonary bypass (CPB) is one of the most groundbreaking medical innovations in history, enabling safe and effective heart surgery by temporarily replacing the function of the heart and lungs. This review starts with ancient concepts of cardiopulmonary function and then traces the evolution of CPB through important physiological and anatomical discoveries, culminating in the development of the modern heart–lung machine. In addition to examining the contributions of significant figures like Galen, Ibn al-Nafis, William Harvey, and John Gibbon, we also examine the ethical and technical challenges faced in the early days of open heart surgery. Modern developments are also discussed, such as miniature extracorporeal systems, off-pump surgical techniques, and the increasing importance of extracorporeal membrane oxygenation (ECMO) and extracorporeal life support (ECLS), while the evolving role of perfusionists in diverse cardiac teams and the variations in global access to CPB technology are also given special attention. We look at recent advancements in CPB, including customized methods, nanotechnology, artificial intelligence-guided perfusion, and organ-on-chip testing, emphasizing CPB’s enduring significance as a technological milestone and a living example of the cooperation of science, medicine, and human inventiveness because it bridges the gap between the past and the future.

## 1. Introduction

The synchronization of the heart and lungs is one of the most significant physiological relationships in the human body, preserving cellular life and systemic homeostasis by continuously and synchronously removing carbon dioxide and supplying oxygen to tissues. Any short-term disruption of this balance could have catastrophic consequences, making it easy to forget the long and complex intellectual process that led to our understanding of cardiopulmonary physiology, even though it forms the basis of much of modern medicine and surgery [1,2,3].

For much of human history, it was unknown how the heart and lungs worked; thus, in their philosophical and symbolic frameworks for comprehending the body, ancient physicians and philosophers regularly relied on observation and speculation. It was believed that the breath (or “pneuma”) was a life force and that the heart was the source of emotion, intellect, or soul. Because they did not have a firm grasp of circulation or gas exchange, early philosophers were unable to mentally differentiate between respiratory and circulatory activities, with the structure of the thoracic cavity being complex but poorly understood, leading to centuries of conjecture and debate [4,5].

Only waves of anatomical research, physiological testing, and technological innovation have allowed us to understand heart–lung dynamics as we do today, paving the way for one of the most groundbreaking medical innovations: cardiopulmonary bypass (CPB). The development of CPB allowed surgeons to temporarily take on the functions of the heart and lungs, enabling the creation of complex open heart surgeries and the treatment of complex cardiac conditions, leading to what began as a conceptual dilemma, becoming the foundation for life-saving interventions [6,7].

Examining the evolution from philosophical speculation to scientific understanding and, ultimately, to technological reality is the aim of this review, emphasizing how the connections between anatomical discoveries, physiological understanding, and surgical technology have shaped our current capabilities. While modern innovations are addressed in detail, this review intentionally devotes substantial attention to the ancient and pre-modern foundations of cardiopulmonary physiology, as these intellectual milestones are integral to understanding the conception and eventual realization of extracorporeal circulation. We hope to shed light on the history of CPB and the greater narrative of human curiosity, experimentation, and perseverance that led to its development by tracing its development from prehistoric theories to Renaissance discoveries to the creation and advancement of the heart–lung machine.

## 2. Materials and Methods

This article integrates interdisciplinary viewpoints from the fields of medicine, physiology, biomedical engineering, and medical ethics in a historical and narrative review format to document the evolution of CPB and related technologies, with the primary and secondary sources that were consulted including historical evaluations, technical reports on CPB, ECMO, and extracorporeal life support systems, modern academic literature, and classical medical works (e.g., Galen, Ibn al-Nafis, and Harvey).

A comprehensive literature search was conducted using databases such as PubMed, Scopus, Google Scholar, and historical archives, with the search terms used including cardiopulmonary bypass, heart–lung machine, extracorporeal circulation, history of heart surgery, ECMO, perfusionist, medical ethics, and surgical innovation. The publication date was not restricted in order to ensure that the most recent clinical and technological developments as well as foundational historical sources were included.

### Inclusion and Exclusion Criteria

Sources were selected according to the following criteria:

Inclusion Criteria:

- Publications that go over the development of cardiopulmonary bypass, extracorporeal circulation, or related physiological and surgical landmarks.
- Peer-reviewed papers, scholarly theses, and monographs that discuss ECMO, CPB, ECLS, or perfusion technologies from a technical, clinical, or ethical perspective.
- Historical medical writings or translations of them (e.g., works by Galen, Harvey, Ibn al-Nafis, Vesalius) had a significant influence on early concepts of cardiac physiology.
- Current studies on the future directions of extracorporeal life support, global availability of CPB, and technological developments.
- Reliable translations of works written in other languages or English-language sources.

Exclusion Criteria:

- Articles that solely covered unrelated surgical specialties or non-cardiopulmonary technologies.
- Studies that have no historical or technological significance for CPB or extracorporeal circulation.
- Sources that are anecdotal or non-scholarly and have no clinical or academic validity.
- Works that are duplicates or derivatives and do not offer any new insights.

As a result, the review was able to preserve its academic caliber and topic coherence while integrating both important historical data and therapeutically pertinent recent discoveries.

When selecting sources, factors like historical significance, relevance, and contribution to the review’s subject structure were taken into account, with the analysis being qualitative in nature in order to integrate technological developments with clinical practice, ethical debates, and global health considerations. Since neither human subjects nor original clinical data were used in this study, ethical approval was not required.

## 3. Ancient Concepts of the Heart and Lungs

### 3.1. Early Greek Speculations

The earliest known remarks on the function of the heart and lungs in Western thought can be found in ancient Greek literature, where physiological ideas were deeply interwoven with myth, philosophy, and poetic metaphor. In Homeric epics, such as the *Iliad* and the *Odyssey*, human life was often described in terms of breath and bodily sensations (8th century BCE), with death being depicted as the departure of the psyche, the vital breath, or soul, while the thymos, believed to be housed in the chest, was associated with emotion, bravery, and consciousness, reflecting an intuitive relationship between breathing, life, and the central thoracic organs despite their lack of anatomical clarity [7,8,9,10].

A more systematic, albeit still speculative, approach was developed by early Greek natural philosophers in the fifth century BCE. Alcmaeon of Croton, often considered a pioneer of modern medicine, was the first to propose that the body functioned through a balance of internal forces and components, with his theory stating that because air, or pneuma, enters the body through breathing and passes through invisible channels to support the brain and other organs, it is vital to health and vitality. Despite his limited understanding of anatomy, Alcmaeon’s conception of pneuma as a life-sustaining force laid the groundwork for later physiological concepts [11,12,13].

The four “roots”—earth, air, fire, and water—later known as the four elements were the subject of a cosmological theory presented in Alcmaeon’s contemporary Empedocles of Acragas, extending this framework into biology by asserting that these fundamental components comprised all living things. His physiological theories state that air (pneuma) enters the body and circulates throughout it due to respiration and blood vessel circulation. Empedocles described the heart as a central mixing chamber where the elemental components interacted, representing a growing understanding of the importance of the heart and the role of air circulation, despite the lack of empirical support [14,15,16].

### 3.2. Aristotle’s Model (4th Century BCE)

One of the most significant figures in ancient natural philosophy, Aristotle (384–322 BCE), developed a comprehensive and systematic knowledge of human anatomy and physiology that had a long-lasting impact on Western medical philosophy. By applying logic, observation, and a teleological framework to explain physical activities, Aristotle expanded on the speculative theories of his predecessors and came to believe that every part of the body had a purpose [17].

According to Aristotle’s paradigm, the heart was the main organ of motion, sensation, and life, believing that it was the first organ to form in the embryo and the seat of intellect, which is now acknowledged to have been based on his observations of chick development. Conversely, the lungs were regarded as passive accessory organs, with their primary responsibility, according to Aristotle’s theory, being to control the heart’s activity and maintain life by reducing the heat that the heart naturally produced, an idea consistent with his broader view of nature as inherently balanced and purposeful [16,18].

Aristotle rejected the notion that the brain is the center of cognition and instead assigned it a cooling function akin to that of the lungs. Additionally, he was unable to imagine a closed circulatory system or the actual mechanics of gas exchange, believing that the blood carried a life force that was transferred by the heart through veins, and he did not comprehend the concepts of continuous circulation or pulmonary transit. He thought that the lungs regulated the body’s internal fire by contracting and expanding and drawing in air to cool the heart [16,19,20].

Despite its anatomical flaws, Aristotle’s physiological theory made sense within the context of ancient Greek philosophy, being among the first to combine anatomical research with a broader metaphysical viewpoint in an attempt to provide a naturalistic explanation of biological processes. His influence continued well into the medieval period, particularly in the scholastic and Islamic medical traditions, and his theories were regularly reconciled with those of Galen and other classical sources.

### 3.3. Hippocratic Corpus

The Hippocratic Corpus shows a more practical and clinical approach to comprehending the human body, whereas Aristotle embodied the philosophical tradition of ancient Greek medicine. The basis for rational medicine in the Western tradition was established by this body of medical treatises, which are traditionally credited to Hippocrates of Kos (c. 460–370 BCE), but were probably written by a number of doctors over time, diverging from mystical explanations of illness by emphasizing methodical observation, prognosis, and the natural causes of disease [21].

The observable functions of the heart and lungs, as well as their roles in preserving the equilibrium of the four humors—blood, phlegm, yellow bile, and black bile—are discussed in the Hippocratic texts. It was believed that disease resulted from imbalance (dyscrasia) and that health was a state of balance (eucrasia) [22]. Pneuma, or the breath, was considered vital to life, and the flow of blood and air within the body was frequently associated with respiration, with some treatises, like On Breaths (Peri Anapnoēs), indicating an awareness of the lungs’ involvement in respiration despite the fact that the mechanism was not well understood and was frequently explained by humoral or elemental theories [23,24].

Although the authors of the Hippocratic Corpus made significant anatomical advances through animal dissection and clinical experience, their knowledge of the thoracic organs was still speculative and lacking [21]. The lungs were recognized as expanding structures connected to the act of breathing, and the heart was described as a warm organ closely associated with emotion and vitality, while not yet understanding the concept of pulmonary circulation or the precise pathways of blood flow [25].

**Figure 1:**
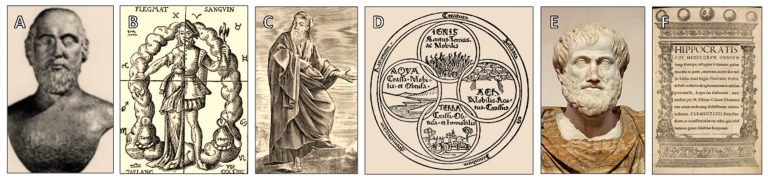
Early Greek medical and philosophical concepts. (**A**) Alcmaeon of Croton (5th century BCE), one of the earliest Greek physician-philosophers to explore the connection between air and life. [Public domain] (**B**) Illustration of the four humors—phlegm, blood, yellow bile, and black bile—linked to bodily health and behavior. These ideas gained prominence through figures like Alcmaeon. Illustration from Quinta Essentia by Leonhart Thurneisser zum Thurn (16th century). [Courtesy: Public domain] (**C**) Empedocles of Akragas (c. 494–434 BCE), who proposed the cosmogonic theory of the four classical elements. [17th-century engraving, public domain] (**D**) Woodcut of Empedocles’ four-element theory (fire, air, water, earth), from a 1472 edition of Lucretius’ De rerum natura. [Public domain] (**E**) Aristotle (384–322 BCE), Greek philosopher and scientist; Roman marble copy of a bronze bust by Lysippos (c. 330 BCE) with modern alabaster mantle. [Public domain] (**F**) First complete Latin edition of the Hippocratic Corpus, Octoginta Volumina (Rome, 1525). [Public domain].

Despite its limitations, the Hippocratic Corpus represented a crucial step toward empirical medicine, displaying dedication to understanding the human body as a system subject to natural laws and to relying on observation rather than conjecture helped create the foundation for later, more precise models of cardiopulmonary function. Furthermore, a fundamental component of contemporary medical practice is the ethical legacy of Hippocratic medicine, emphasizing the doctor’s duty to “do no harm” [26]. Along with illustrations of the four elements and humoral theory, Figure 1 highlights important early Greek contributions to medical thought, such as Alcmaeon, Empedocles, Aristotle, and the Hippocratic tradition. Early Greek and Hippocratic ideas of pneuma and circulation, though speculative, established enduring frameworks linking breath, blood, and vitality. These concepts provided the intellectual scaffolding on which later anatomical and physiological discoveries would build.

## 4. Galenic Physiology and Its Dominance

### 4.1. Galen’s Cardiopulmonary Model (2nd Century CE)

Galen of Pergamon (c. 129–c. 216 CE) had a lasting and significant impact on medical history, with Galen, a prolific writer and physician to Roman emperors, combining and developing earlier Greek medical theories, particularly those of Hippocrates and Aristotle, into a systematic and comprehensive body of knowledge that would shape medical thought for more than a thousand years [27].

Since human dissection was typically prohibited in the Roman Empire, Galen’s knowledge of the heart and lungs was based on animal dissections, especially those of pigs and apes, with his theoretical framework being remarkably coherent, despite being limited by anatomical errors. According to Galen, venous blood from the body entered the right side of the heart and was subsequently sent to the lungs via the pulmonary artery. Lacking an understanding of pulmonary circulation, he proposed that some of this blood traveled straight from the right ventricle to the left via microscopic, imperceptible holes in the interventricular septum, a theory that would stand the test of time for centuries [28,29,30,31].

According to Galen’s model, the lungs had a secondary function in altering the blood rather than being the location of vital gas exchange. According to his theory, air, or pneuma, enters the body through the lungs and refines and vitalizes the blood after being absorbed, with the left ventricle sending this newly changed blood, now containing pneuma, through the aorta to feed and maintain the body’s processes. Additionally, Galen believed that the venous system transported a different, more nutrient-dense form of blood, while the arterial system carried this spirit-enriched blood [32,33].

In spite of his model’s flaws, most notably, the absence of interventricular pores and the disregard for pulmonary circulation, Galen’s framework made sense given the limitations of his resources and time, being so thorough and authoritative that they became the accepted standard of reference in both medieval Europe and the Islamic Golden Age. His theories aligned with the dominant humoral and Aristotelian theories of physiology, thus successfully delaying the correction of his mistakes until the Scientific Revolution, several centuries later, by combining philosophy, anatomy, and clinical practice [34,35].

### 4.2. Influence of Galenic Thought

Galenic physiology’s supremacy in both the Islamic and Western worlds was due to its comprehensiveness, philosophical sophistication, and conformity to dominant medical and metaphysical worldviews, not just its internal coherence. For more than 1300 years, academics, medical professionals, and educators alike held Galen’s writings in almost religious awe as the epitome of anatomical and physiological knowledge [36].

Galen’s writings were widely translated, preserved, and discussed during the Byzantine and Islamic Golden Ages, where Galenic models were not only adopted but also integrated into larger philosophical and medical systems by Islamic scholars like Avicenna (Ibn Sina) [37] and Averroes (Ibn Rushd) [38]. In the 11th and 12th centuries, translations from Arabic brought the Galenic corpus back to Latin-speaking Europe, where it served as the basis for the curricula of recently founded medieval medical schools [36].

There were substantial scientific costs associated with this long-standing adherence to Galenic doctrine. For generations, his dual-blood system (venous vs. arterial), his theory of porous interventricular septa, and his misinterpretation of the lung’s function in gas exchange were accepted as facts. Dissection, when allowed, was frequently used to validate Galenic anatomy rather than test anatomical theories because the authority of Galen’s teachings discouraged empirical inquiry, with disparities between Galen’s descriptions and actual observations being frequently written off as anatomical anomalies rather than theoretical mistakes [34,39].

This intellectual inertia directly contributes to the delay in pulmonary circulation recognition, with scholars like Ibn al-Nafis, Servetus, and Harvey eventually producing brilliant insights. However, because they went against Galenic orthodoxy, they were slow to be accepted [33,40].

Galen’s influence was not wholly obstructive, however, causing for centuries, medical education and practice to be influenced by his emphasis on observation, his creation of clinical methodology, and his fusion of philosophy and medicine. Despite its shortcomings, the Galenic tradition laid the groundwork for modern medicine when scientific research became a vital component again during the Scientific Revolution and the Renaissance [30,32]. Galen’s model, despite inaccuracies, dominated medical teaching for over a millennium. Its preservation and dissemination through Byzantine, Islamic, and Western traditions ensured that circulation remained a central focus, setting the stage for eventual challenges and refinements.

## 5. Renaissance and Scientific Revolution: Challenging Ancient Theories

### 5.1. Discovery of Pulmonary Circulation

Modern physiology did not emerge quickly or in a straight line from medieval adherence to Galenic models, but rather developed as a result of the efforts of lone academics who started challenging long-held beliefs, frequently alone and at considerable personal risk. The understanding that blood moves from the heart’s right ventricle to the lungs, where it changes, and then on to the left ventricle for systemic distribution, was one of the most important discoveries, ultimately destroying Galen’s porous interventricular septum model and permanently change our knowledge of cardiopulmonary physiology [33,36,39].

Ibn al-Nafis (1213–1288), a Syrian physician from the 13th century, is credited with articulating this concept in the earliest known way, with his writings marking a significant break from Galenic orthodoxy. Ibn al-Nafis contended in his commentary on Avicenna’s writings that blood is not transported through the septum between the right and left ventricles, but rather is driven from the right ventricle into the lungs by the pulmonary artery, where it interacts with air, and then returned to the left ventricle by the pulmonary vein. In advance of subsequent anatomical discoveries, he also described the thinness of the pulmonary capillaries and the porous nature of the lung’s tissue. It is noteworthy that his explanation of pulmonary circulation is not only conceptually correct but also backed by a resounding denial of Galenic doctrine [41,42].

Even though Ibn al-Nafis’s insights were brilliant, their immediate historical impact was limited because his work was not well-known in Europe until the 20th century, but eventually thanks to the work of Spanish theologian, physician, and polymath Miguel Servet (Michael Servetus), the same idea reappeared in the 16th century. Servet discussed the flow of blood through the lungs as part of a larger discussion about the spirit and the soul in his theological treatise Christianismi Restitutio, published in 1553. He outlined the key components of pulmonary circulation by saying that blood is sent from the heart to the lungs, where it is combined with air before returning to the heart, representing an independent rediscovery of the pathway outlined by Ibn al-Nafis, despite not being exclusively anatomical in nature [43,44].

Unfortunately, Servet’s religious writings, which were deemed heretical, overshadowed his scientific contributions, with his books being destroyed when he was arrested in Geneva and executed by burning at the stake in 1553, another instance of how scientific truth has frequently surfaced beneath the specter of ideological conflict [43,44].

**Figure 2:**
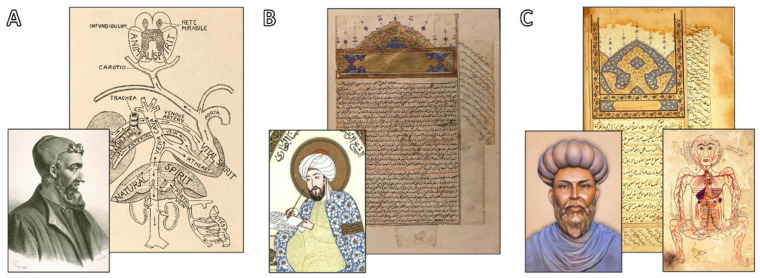
Classical and medieval contributors to cardiopulmonary thought. (**A**) Galen of Pergamon (129–c. 216 CE), whose physiological theories dominated medicine for over a millennium. Lithographic portrait by Pierre Roche Vigneron; diagram illustrating Galen’s physiological model. [Courtesy: Wellcome Collection] (**B**) Avicenna (980–1037), Persian polymath and author of The Canon of Medicine. First page of a 1597/98 manuscript. [Courtesy: Yale University Library] (**C**) Ibn al-Nafis (1210/11–1288/89), who first accurately described pulmonary circulation in the 13th century. Opening page of one of his medical texts, likely copied in 17th–18th century India. [Public domain].

Ibn al-Nafis and Miguel Servet’s combined insights mark a significant departure from the long-standing mistakes of Galenic physiology. From antiquity through the Islamic Golden Age, medical knowledge was shaped by the models of cardiopulmonary function developed by Galen, Avicenna, and Ibn al-Nafis, as shown in Figure 2, preparing the way for William Harvey’s conclusive formulations of cardiopulmonary dynamics in the 17th century, but it would still take decades for their theories to become part of the mainstream of medical knowledge.

### 5.2. William Harvey’s Discovery (1628)

The groundbreaking work of English physician and anatomist William Harvey (1578–1657) ultimately overthrew the centuries-old structure of Galenic physiology in the early 17th century. Harvey’s seminal work, Exercitatio Anatomica de Motu Cordis et Sanguinis in Animalibus (An Anatomical Exercise on the Motion of the Heart and Blood in Animals), also known as De Motu Cordis, was published in 1628, with the understanding of cardiovascular function being forever altered when Harvey provided the first systematic and evidence-based description of a closed circulatory system in this succinct but incredibly influential work [45].

Harvey proved that the heart worked as a muscular pump that pumped blood through the body in a circular motion through painstaking animal experimentation, vivisection, and mathematical reasoning, presenting strong evidence that blood flowed continuously in a single direction, from the heart to the periphery and back again, rather than ebbing and flowing as Galen had claimed. He determined that if Galen’s model were accurate, the volume of blood flowing through the heart would greatly exceed the volume of blood in the body, demonstrating that recirculation was not only conceivable but also required [45,46].

Despite Harvey’s description of the lungs as a component of this circulatory system, it was still unclear exactly what oxygenation did, while the work of Antoine Lavoisier in the 18th century, discovered the existence and purpose of oxygen as a separate element. Harvey, however, correctly mapped the pulmonary transit of blood from the right ventricle to the lungs and then to the left ventricle after noticing that blood changed color as it moved through the lungs. However, because he did not have a microscope, he was unable to see capillaries, which remained the missing component of his model until Marcello Malpighi described them decades later [44,47,48].

Harvey’s contributions signaled a change in perspective, by promoting experimental observation and mathematical reasoning over inherited dogma, which was not only a methodological breakthrough but also a victory of anatomical and physiological insight. Despite initial skepticism, especially from those who supported the Galenic tradition, Harvey’s discoveries eventually became widely accepted and established the foundation for contemporary cardiovascular physiology [44,49].

Importantly, his model created a mechanical understanding of the heart and circulation that would serve as the foundation for CPB technology centuries later, laying the groundwork for interventions that would alter or temporarily replace these natural processes by viewing the heart as a pump and circulation as a closed system, which are fundamental to the concept of extracorporeal circulation. Regarding the impact on CPB development, breakthroughs by Ibn al-Nafis, Servetus, and Harvey replaced Galenic models with more accurate understandings of pulmonary and systemic circulation. These advances created the conceptual precondition for the later mechanization of circulation outside the body.

## 6. Advances in Thoracic Anatomy and Physiology (17th–19th Centuries)

### 6.1. Anatomical Refinement

Anatomical knowledge advanced significantly in the years after Harvey’s groundbreaking work, driven by the increasing recognition of dissection and empirical observation as valid scientific methods. Anatomists and physiologists, who were committed to closely examining and recording the human body’s structures with previously unheard-of accuracy and rigor, gradually supplanted Galenic authority during these centuries [44].

Andreas Vesalius (1514–1564), one of the pioneers and most significant figures in this movement, contributed to the anatomical revolution with his work, which came a little before Harvey’s. By directly dissecting human cadavers, Vesalius’ painstakingly illustrated anatomical atlas De humani corporis fabrica (On the Fabric of the Human Body), published in 1543, fixed many of Galen’s mistakes. Vesalius changed the study of human anatomy by emphasizing firsthand observation over blind textual reverence, even though he kept some Galenic ideas, establishing a new benchmark for anatomical precision and prepared the way for later anatomist generations to delve deeper into the study of thoracic structures [50,51].

The work of Italian anatomist and microscopist Marcello Malpighi (1628–1694) in the 17th century marked a significant breakthrough. Malpighi employed one of the first microscopes to view the intricate details of living tissues, expanding upon Harvey’s circulation model, being the first to characterize the network of microscopic vessels that linked the lungs’ arteries and veins, known as the pulmonary capillary beds, in 1661. By establishing the hitherto unnoticed connection between arterial and venous blood flow, this finding completed Harvey’s circulatory model [52].

A significant development in microanatomy was Malpighi’s discovery of capillaries, which demonstrated that blood passed through the lungs via a delicate and ongoing vascular network. In addition to supporting Harvey’s theory, his observations helped to clarify that the lungs are active organs of exchange rather than passive blood modifiers, with later findings in respiratory physiology further clarifying the idea that the lungs were places where blood and air came into close contact [53,54].

**Figure 3:**
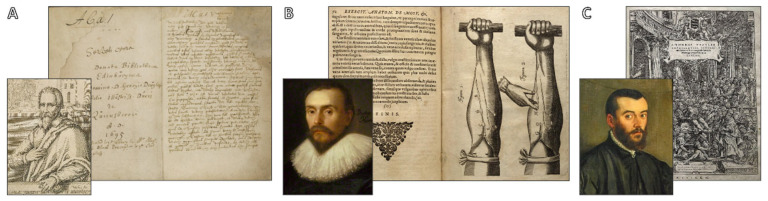
Renaissance and scientific revolution milestones. (**A**) Michael Servetus (1511–1553), Spanish physician and theologian who described pulmonary circulation in Christianismi Restitutio (1553). [Portrait: Amsterdam, 1607; Courtesy: University of Edinburgh] (**B**) William Harvey (1578–1657), who discovered systemic circulation, shown in a 1627 portrait by Daniël Mijtens. Title page of De Motu Cordis (1628). [Courtesy: National Portrait Gallery, London] (**C**) Andreas Vesalius (1514–1564), pioneer of modern anatomy. Portrait by Jan van Calcar; cover of De Humani Corporis Fabrica (1543). [Courtesy: Hermitage Museum].

An even more sophisticated understanding of thoracic anatomy was made possible by developments in anatomical illustration, preservation techniques, and dissection techniques by the 18th and 19th centuries. Servetus, Harvey, and Vesalius are shown in Figure 3, with their work reinterpreting the scientific method of studying human anatomy and circulation and disproved Galenic doctrines. Atlases like those by William Hunter, Henry Gray, and others became indispensable teaching aids as medical schools throughout Europe started to place a strong emphasis on systematic anatomical training [55]. All of these advancements combined to create a more precise and thorough map of the heart, lungs, and related vasculature, information that is essential for the development of mechanical circulatory support systems in the future.

### 6.2. Development of Respiratory Physiology

The structural basis for comprehending the cardiopulmonary system was established by anatomical advancements, but the expanding field of physiology, specifically, the study of respiration, offered vital information about the lungs’ functional role in sustaining life. A number of experimental discoveries between the 17th and 19th centuries gave rise to a concrete, chemical understanding of gas exchange and oxygen transport, replacing the abstract ideas of pneuma and elemental balance [4,30].

The work of French chemist Antoine Lavoisier (1743–1794), who is frequently referred to as the father of modern chemistry, marked a significant turning point in the late 18th century, proving that air was a mixture of gases, including the recently discovered element oxygen, rather than a single, life-giving substance as was previously believed. He learned that oxygen was necessary for respiration and combustion and that it reacted with organic materials in the body to create energy, releasing carbon dioxide as a byproduct in the process, making a revolutionary connection between breathing and cellular metabolism by redefining respiration as a chemical process similar to slow combustion [56,57].

Previous gas analysis experiments, especially those conducted by Joseph Priestley and Carl Wilhelm Scheele, who separately isolated oxygen, enabled Lavoisier’s discoveries. Lavoisier, however, was the first to quantify the gas exchange during respiration and to conceptualize the lungs as the location where carbon dioxide was expelled and oxygen entered the bloodstream, demonstrating the lungs’ functional role in maintaining life through a quantifiable, chemical interaction, despite his lack of understanding of the molecular mechanism of gas transport [56,58].

Lavoisier laid the groundwork for later respiratory physiology research. John Dalton developed the law of partial pressures in the 19th century, and Christian Bohr subsequently explained the Bohr effect, which is the connection between the concentration of carbon dioxide and the release of oxygen from hemoglobin, providing more information about the oxygenation process that takes place during pulmonary circulation and clarified the dynamic equilibrium between gases in the blood and the lungs [59,60].

**Figure 4:**
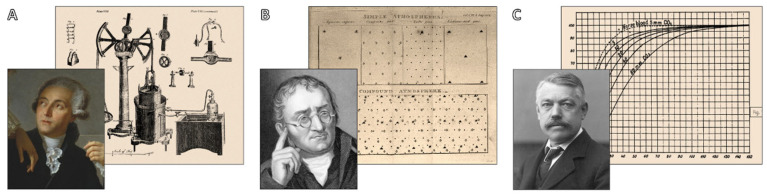
Advances in respiratory physiology and gas exchange. (**A**) Antoine-Laurent de Lavoisier (1743–1794), who identified the role of oxygen in respiration. Detail from Jacques-Louis David’s portrait and an engraving of his gas collection apparatus. [Courtesy: Metropolitan Museum of Art] (**B**) John Dalton (1766–1844), known for Dalton’s Law of partial pressures. Portrait by Zanetti and Agnew (1823); diagram from his essay on gas constitution and evaporation. [Courtesy: Science Museum Group & Wellcome Collection, CC-BY] (**C**) Christian Bohr (1855–1911), who developed the Bohr effect. Oxygen dissociation curves demonstrating PCO_2_ influence on hemoglobin affinity for oxygen. [Public domain].

By the end of the 19th century, a complete picture had developed: the heart functioned as a pump to move the blood through a closed, continuous circuit, while the lungs acted as a finely tuned interface where blood absorbed oxygen and released carbon dioxide. Lavoisier, Dalton, and Bohr are shown in Figure 4; they all made contributions to our growing knowledge of respiration, gas exchange, and oxygen transport, with the development of artificial systems to mimic or temporarily replacing the function of the heart and lungs was made possible by this expanding body of physiological knowledge, thus helping pave the way for the development of CPB technology in the 20th century. The era’s integration of microscopy, respiratory chemistry, and experimental physiology clarified the mechanics of gas exchange and blood flow. These discoveries provided the technical knowledge essential for designing extracorporeal circuits in the 20th century.

### 6.3. Early Extravascular Perfusion and Oxygenation Experiments (1812–1930s)

#### 6.3.1. Conceptual Origin

In 1812, César-Julien-Jean Le Gallois hypothesized that life might be sustained by an apparatus capable of maintaining circulation and gas exchange outside the body—an early conceptual sketch of extracorporeal circulation that foreshadowed later technical developments [61].

#### 6.3.2. Organ Perfusion Prototypes

By the mid-19th century, physiologists were building closed circuits to perfuse isolated organs. Ernst Bidder’s 1862 apparatus for renal perfusion is an early example of a purpose-built system, illustrating both the feasibility and the experimental challenges of sustaining organ function ex vivo [62].

#### 6.3.3. Toward Artificial Oxygenation and Closed Circuits

In the 1880s, European investigators developed devices that explicitly combined pumping with gas exchange. Frey and Gruber (1885) constructed a pulsatile pump with an “artificial lung” (oxygenator) within a closed circulation loop—an important conceptual step toward modern pump oxygenators. In the following decade, Carl Jacobj introduced his “Hämatinator” (1890), a water-driven two-valve pump with explicit provision for oxygenation; he and collaborators also explored a “double Hämatinator” using a biological lung to avoid direct air–blood contact. These experiments seeded three classic extracorporeal oxygenation strategies—bubble, film, and isolated lung—that would ultimately inform clinical devices half a century later [62,63].

#### 6.3.4. Pre-Clinical Heart–Lung Machines

Between the World Wars, Sergei Brukhonenko’s group in Moscow built and tested one of the first devices approximating a heart–lung machine (“autojector”), performing total-body perfusions in canines and reporting the work not only in Russian but also in French-language journals beginning in 1928–1929—an important, if under-recognized, strand in the pre-Gibbon evolution of extracorporeal support [64,65,66].

#### 6.3.5. From Gas Dispersion to Clinical Translation (1950–1955)

Leland C. Clark, Jr. and colleagues demonstrated oxygenation of blood by gas dispersion (1950) and catalyzed the adoption of antifoam strategies crucial for bubble/disc oxygenators—developments that materially reduced foam-related complications and helped bring extracorporeal oxygenation into the operating room. Soon thereafter, A. M. Dogliotti reported the first human use of an apparatus for extracorporeal blood circulation (1951). In the same year, Clarence Dennis performed the first CPB-assisted cardiac operation (an attempted ASD repair), inaugurating the clinical era despite an initial fatal outcome; these early efforts rapidly converged with subsequent successes. Finally, C. Walton Lillehei’s cross-circulation (1954–1955) provided a transformative bridge technique that enabled definitive open heart repairs at scale while pump oxygenators matured [67,68,69,70].

#### 6.3.6. Impact on CPB Development

Collectively, these 19th- and early 20th-century advances established the core design grammar of extracorporeal support—closed circuits, pulsatile or continuous pumping, and engineered gas exchange—while also surfacing safety issues (hemolysis, foam, air–blood contact) that would shape later materials science and biocompatibility. They formed the practical and conceptual runway upon which Gibbon and contemporaries could achieve sustained cardiopulmonary bypass in humans [63,71].

## 7. Early Attempts at Mechanical Heart and Lung Support

### 7.1. Early Cardiac Surgery Challenges

How to surgically intervene on a beating heart without causing fatal circulatory arrest became a critical new challenge as our understanding of the anatomy and physiology of the heart and lungs developed in the 19th and early 20th centuries, with the heart remaining largely inaccessible because of its constant motion and the absolute necessity of uninterrupted circulation and oxygenation, even though advancements in anesthesia, antisepsis, and operative techniques were revolutionizing many areas of surgery [72].

Early cardiac surgeons faced a difficult conundrum. In order to operate on the heart, it had to be stopped, but this would stop oxygenated blood from flowing, which would cause rapid, irreversible brain damage and death. Without a way to temporarily take over the functions of both the heart and the lungs, even basic repairs, like removing obstructions or closing congenital defects, could not be safely carried out [73,74,75].

Several daring and experimental methods were investigated in order to address this conundrum, with pioneering doctors experimenting with the concept of extracorporeal circulation in the late 19th and early 20th centuries, involving pumping the patient’s blood through external devices to sustain life while the heart is stopped. However, at this point, there was no technology in place to safely and carefully pump blood and oxygenate it outside the body [76].

Cross-circulation experiments, in which a second individual, typically a parent in pediatric cases, acted as a “living heart–lung machine,” were among the more audacious inventions. The patient’s blood was transferred into the donor’s circulatory system, where it was filtered and oxygenated by the donor’s lungs before being given back to the patient, producing some early surgical successes in the 1950s, especially in the hands of C. Walton Lillehei, despite being morally and practically challenging, made evident nevertheless that a mechanical substitute was required, one that could consistently sustain oxygenation and circulation without requiring the use of an additional human body [77,78].

### 7.2. Pre-CPB Innovations

The foundation for CPB was established by several experimental prototypes and conceptual advances prior to the successful development of a fully operational heart–lung machine, arousing at the nexus of biology, engineering, and medicine as scientists and surgeons worked to create systems that could sustain circulation and gas exchange outside of the human body [76,77].

One of the most innovative early attempts was made by French surgeon and Nobel laureate Alexis Carrel, who worked with American inventor and aviator Charles Lindbergh to create a perfusion pump in the 1930s. The Carrel–Lindbergh apparatus, a sealed, sterile system that maintained blood or nutrient solutions flowing through isolated organs for prolonged periods of time, was inspired by the goal of maintaining organs outside the body for research and transplantation. Despite not being designed for human use, this device made a significant conceptual advancement toward extracorporeal life support by demonstrating that organ function could be maintained ex vivo [79,80].

Simultaneously, scientists were investigating various techniques for externally oxygenating blood, with the problem being twofold: oxygen had to be moved from air to blood efficiently without creating dangerous bubbles (air embolism), and blood had to be pumped efficiently without causing cell damage (hemolysis). In an effort to maximize surface area for gas exchange while reducing damage to blood components, early designs used film oxygenators, rotating discs, and bubble oxygenators [81,82].

The work of John Gibbon, an American surgeon who envisioned a machine that could take over both heart and lung functions during cardiac surgery, marked a significant milestone in the 1940s. Although the results of his early animal experiments were not entirely satisfactory, they did yield important information on flow dynamics, oxygenation, and anticoagulation, using a vertical screen oxygenator and a roller pump. Over the course of more than ten years of work, he gradually improved the system in spite of technical difficulties [83].

Although these pre-CPB innovations were not yet prepared for routine clinical use, they reflected a growing body of scientific evidence that mechanical support of the cardiopulmonary system was both theoretically feasible and becoming more accessible, with pumps, oxygenators, and closed circuits, the essential parts of contemporary CPB, being all in the experimental stage, waiting for the discoveries that would soon enable their use in human applications.

## 8. The Development of the Cardiopulmonary Bypass Machine

### 8.1. Key Figures

One of the most revolutionary developments in the history of biomedical engineering and surgery is the development of a working CPB machine, with the heart–lung machine’s father, Dr. John Heysham Gibbon (1903–1973), being widely credited with having the vision and perseverance that led to this breakthrough. Operating on a non-beating heart was once unimaginable, but thanks to his decades of dedication, it is now a clinical reality [83].

A very personal clinical experience in the early 1930s, when he saw a young woman die from a massive pulmonary embolism, served as Gibbon’s inspiration, thinking he might have saved her life if he could have temporarily bypassed her heart and lungs. The search for a device that could oxygenate and circulate blood outside the body during surgery was sparked by this tragic incident and lasted for a lifetime [84,85].

Using money from the American Philosophical Society and the help of his wife, Mary Gibbon, and IBM engineers, Gibbon worked for years to perfect a machine that exposed blood to oxygen using a film oxygenator and a roller pump. In 1953, he accomplished a historic first: the successful use of a heart–lung machine during open heart surgery, following extensive animal testing. He fixed an 18-year-old patient’s atrial septal defect during this procedure, and the patient recovered completely, marking the first time in medical history that a human heart was surgically repaired with complete machine-assisted circulation and oxygenation [86].

Gibbon’s success spurred a wave of advancements, despite the fact that his original machine was complicated and not widely used in its original form. Through changes that improved its dependability, portability, and clinical applicability, surgeons like C. Walton Lillehei, John Kirklin, and Richard DeWall quickly advanced CPB technology. Particularly significant were DeWall’s invention of the bubble oxygenator and Lillehei’s use of cross-circulation as a transition to complete CPB. Concurrently, Kirklin at the Mayo Clinic greatly increased the clinical acceptance of CPB by standardizing its use in congenital heart surgery [83,85,87,88].

Along with overcoming difficult technical challenges like hemolysis, air embolism, and blood clotting, these trailblazers also faced moral dilemmas related to the dangers of experimental surgery, setting the stage for what would eventually become a common practice in heart surgery across the globe.

**Figure 5:**
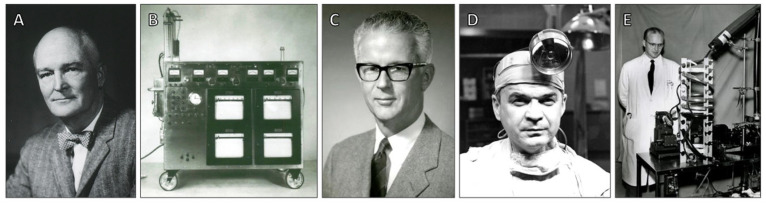
Pioneers of modern cardiopulmonary bypass. (**A**) John H. Gibbon (1903–1973), inventor of the heart–lung machine. [Public domain] (**B**) Gibbon’s original heart–lung machine, developed in collaboration with IBM. [Courtesy: Thomas Jefferson University Archives, Philadelphia] (**C**) John W. Kirklin (1917–2004), who refined Gibbon’s design for clinical use. [Courtesy: UAB Archives, University of Alabama at Birmingham] (**D**) Clarence Walton Lillehei (1918–1999), a pioneer of open heart surgery and perfusion techniques. [Photo credit: University of Minnesota] (**E**) Richard A. DeWall (1926–2016), who created the first reliable bubble oxygenator to reduce air embolism. Photo of DeWall and the device (1956). [Courtesy: University of Minnesota].

By the end of the 1950s, the heart–lung machine had evolved from an experimental device to a useful and increasingly secure instrument that routinely performed previously unfeasible procedures, having changed the course of modern medicine and saved many lives in the decades that followed by ushering in the age of open heart surgery. Key cardiopulmonary bypass pioneers Gibbon, Kirklin, Lillehei, and DeWall are highlighted in Figure 5 for their contributions that made open heart surgery a clinical reality.

### 8.2. Principles of Cardiopulmonary Bypass

CPB’s primary purpose is to momentarily take on the functions of the heart and lungs so that surgeons can perform controlled surgery on a heart that is immobile and bloodless, being accomplished by rerouting the patient’s blood via an extracorporeal circuit that sustains constant systemic circulation, carbon dioxide elimination, oxygenation, and temperature control. Despite the significant advancements in technology since its inception in the 1950s, the fundamental ideas of CPB have remained remarkably stable [76,89].

A typical CPB procedure involves draining venous blood from the body and directing it into a venous reservoir, usually through the right atrium or the superior and inferior vena cava. The blood is then forced forward under regulated pressure by a roller or centrifugal pump, then going through an oxygenator, which exposes it to a regulated gas mixture that is usually low in carbon dioxide and high in oxygen. Gas exchange mimics the lungs’ alveolar function by passing through a semipermeable membrane or, in older designs, by bubbling [89,90].

After passing through the oxygenator, the blood is oxygenated and its carbon dioxide content is reduced to physiological levels (i.e., toward normocapnia, as achieved by modern membrane oxygenators). Then, the blood is filtered, heated, and then sent back to the body via the aorta or a major artery, avoiding the heart and lungs, with anticoagulation, typically with heparin, being necessary throughout the procedure to stop clots from forming in the circuit. To increase safety and accuracy, modern machines also have automated control systems, bubble detectors, pressure sensors, and air traps [77,89,91].

The system’s biological compatibility and mechanical performance are both critical to CPB’s success. Because blood is a sensitive tissue, coming into contact with synthetic surfaces can set off a series of coagulative, hemolytic, and inflammatory reactions, being considerably lessened by developments in materials science, especially the creation of biocompatible coatings and closed-circuit designs [81]. Numerous procedures have been made possible by CPB, including the following:

- Repairs for congenital cardiac defects.
- Repairs and replacements for cardiac valves.
- Coronary artery bypass grafting (CABG).
- Complex thoracic and aortic procedures.

The field of thoracic and cardiac surgery has been transformed by the ability to precisely control the physiological environment during surgery, maintain systemic perfusion, and safely arrest the heart, making it a shining example of interdisciplinary innovation when engineering, physiology, and surgical techniques come together to preserve life in the most trying circumstances [81,91].

## 9. Ethical and Clinical Implications of Cardiopulmonary Bypass

### 9.1. Ethical Challenges

In addition to being a surgical and technical advance, the development of CPB in the middle of the 20th century also marked a period of significant ethical ambiguity. CPB was a high-risk, experimental intervention in its early clinical uses, frequently administered to patients who had no other treatment options and life-threatening conditions, calling in question the ethical obligations of doctors stepping into uncharted territory, the limits of experimental surgery, and the balance between benefit and harm [76,77].

Significant morbidity and mortality were associated with the initial applications of CPB. Many early patients did not survive because of the intricacy of the equipment, the novelty of the procedures, and the incomplete knowledge of extracorporeal physiology. Even after John Gibbon’s first successful case in 1953, he gave up on further developing the technology because his subsequent attempts had unsatisfactory results. Driven by the urgent need to save lives, particularly in children with congenital heart disease, and the hope of long-term surgical benefit, other pioneers, like C. Walton Lillehei, persisted despite high complication rates [77,81], bringing up challenging moral dilemmas:

- Was exposing patients, particularly young ones, to unproven technologies with unknown results justified?
- Given that neither patients nor doctors fully understood the risks, how should informed consent be obtained?
- Who was morally accountable for deaths brought on by experimental surgery?

By today’s standards, informed consent was either nonexistent or very basic in many early cases, and even in the absence of solid information or complete patient understanding, doctors frequently made decisions based on therapeutic paternalism, acting in what they thought was best for the patient. Furthermore, early CPB research frequently used animal testing and inventions created without official ethical review boards, which would later become commonplace in clinical research [92,93,94].

Despite these reservations, hesitancy was frequently outweighed by the clinical necessity, especially in pediatric cases, with CPB providing the only chance of survival for many patients despite its experimental nature. CPB changed from being a controversial invention to a widely used, life-saving tool as methods advanced and results stabilized over time, but its early history serves as a potent reminder of the moral dilemmas that arise with medical advancement [95].

Modern medical ethics, such as the formalization of clinical trial protocols, informed consent procedures, and institutional review boards (IRBs), were sparked by the legacy of these difficulties, with CPB pioneers influencing an evolving framework that aims to strike a balance between innovation and patient safety and autonomy, a balance that is still crucial in the quickly changing medical landscape of today.

### 9.2. Clinical Breakthroughs

The introduction and development of CPB swiftly produced some of the most important clinical advances in medical history, despite the early ethical and technical challenges. A new era of cardiac and thoracic surgery was ushered in by the ability to temporarily replace the function of the heart and lungs during surgery in the late 1950s and early 1960s, turning previously unfeasible procedures into safe and increasingly common interventions [76,81].

Correcting congenital heart defects was one of the first and most significant uses of CPB. Conditions like tetralogy of Fallot, transposition of the great arteries, and ventricular and atrial septal defects were frequently fatal in infancy or childhood before the development of bypass technology. Previously unthinkable, CPB allowed surgeons to see and fix these flaws inside the open, stationary heart. As methods advanced, survival rates increased steadily, giving newborns with potentially fatal cardiac defects new hope [96,97,98].

Additionally, CPB made it possible to safely perform valvular heart surgery, such as replacing the aortic and mitral valves, which were previously thought to be too risky because they required precise intracardiac manipulation. Likewise, the procedure paved the way for the creation of coronary artery bypass grafting (CABG), a key component of ischemic heart disease treatment. The success of CABG in the 1970s and 1980s made CPB a key part of adult heart surgery and helped to drastically lower the death rate from heart attacks [81,99].

In addition to the heart, CPB played a key role in the development of thoracic and vascular surgery by facilitating intricate operations on the pulmonary arteries, great vessels, and thoracic aorta. It was also a vital auxiliary in heart transplantation and procedures involving circulatory arrest. It also played a part in emergency situations, such as massive pulmonary embolism and cardiopulmonary resuscitation, where it temporarily maintained life during otherwise fatal situations [90,100,101].

With steadily declining complication rates, CPB also became safer and more accessible as results and technology improved. The procedure became flexible for a variety of patients, from newborns to the elderly, thanks to advancements in perfusion techniques, blood conservation, anticoagulation management, and patient monitoring [77].

It is hard to overestimate CPB’s clinical impact. It expanded the range of surgical possibilities, allowing doctors to treat and even cure diseases that were previously always fatal. The ability to safely stop and restart the heart and replace its function with a machine created by human ingenuity has saved and significantly improved millions of lives. What started out as a dangerous and morally dubious experiment has developed into one of the most effective instruments in contemporary medicine [102].

## 10. Modern Advances and Alternatives

The need to improve surgical results, lower physiological stress, and increase patient safety has led to a remarkable evolution in CPB technology in recent decades, having been refined into smaller, smarter, and more biocompatible platforms by advances in engineering, materials science, and data integration, thus making it possible to perform increasingly complex procedures with fewer complications [102].

### 10.1. Miniaturization of Cardiopulmonary Bypass

The creation of minimal extracorporeal circulation (MECC) systems is among the most revolutionary developments in the evolution of CPB, being a new generation of extracorporeal support that emphasizes minimal invasiveness, hemodynamic stability, and biocompatibility in order to lessen the physiological burden of traditional CPB [103,104,105].

In contrast to conventional CPB circuits, MECC systems function as closed-loop circuits that use substantially less priming volume, eliminate the open venous reservoir, and decrease the blood–air interface. Hemodilution, inflammatory mediator release, complement activation, and coagulation disturbances, all major causes of postoperative complications in traditional CPB, are greatly reduced by the lack of a cardiotomy reservoir and the incorporation of a small, heparin-coated circuit, and in certain patients, demonstrating an to decrease myocardial damage, postoperative bleeding, renal dysfunction, and systemic inflammatory response syndrome (SIRS) [106,107,108,109,110,111,112]. Contemporary evidence synthesizing randomized trials and controlled studies indicates that MiECC is associated with reduced postoperative morbidity (including transfusion and inflammatory complications) and may contribute to lower mortality compared with conventional CPB, supporting broader adoption where teams have appropriate expertise [103]. Compared with conventional CPB, MiECC has been shown in meta-analyses to significantly reduce transfusion requirements, ICU stay, and postoperative complications, though long-term survival benefits remain less clear, highlighting the importance of case selection and institutional experience [113].

MECC systems’ small size and straightforward technical design make them perfect for minimally invasive cardiac procedures such as valve surgeries, off-pump-like CABG, and reoperations with limited operative field and tissue damage. Additionally, because of their physiological advantages, they are especially beneficial for high-risk groups that are more susceptible to the negative consequences of traditional bypass surgery, such as elderly patients, patients with ventricular dysfunction, or patients with coagulopathies and multiple comorbidities [105,108,112].

Nevertheless, there are costs associated with MECC’s advantages, with traditional CPB’s buffering ability being lost when a venous reservoir is removed, necessitating careful volume control and continuous hemodynamic monitoring. The circuit may be less forgiving in situations involving abrupt volume changes or intraoperative bleeding, and air handling and suction integration need to be strictly regulated, resulting in MECC working best in carefully chosen patient cohorts and by surgical teams knowledgeable about its physiology and limitations [106,107,108].

Notwithstanding these factors, mounting clinical data indicates that MECC is either superior or non-inferior in terms of fewer transfusion requirements, shorter intensive care unit stays, and quicker postoperative recovery, with ongoing advancements seeking to hybridize MECC with conventional CPB By fusing the biological benefits of closed-circuit circulation with the safety of open systems. Additionally, MECC is a prime example of the transition from a “one-size-fits-all” approach to precision extracorporeal support catered to each patient’s risk profile and surgical requirements as part of the larger trend toward patient-specific perfusion strategies [104,110,111].

In this situation, MECC is more than just a technical improvement; it signifies a philosophical change in the application of CPB, moving from maximal support to minimal intrusion and from physiological compromise to physiological preservation. The broad use of MECC has remained restricted and, in certain areas, has even decreased recently, despite its physiological benefits and encouraging results, caused by a number of factors. First, MECC is less forgiving in complex or high-bleeding-risk surgeries due to its technical requirements and learning curve, especially its requirement for precise volume control, limited suction capacity, and lack of reservoir flexibility, thus making many surgical teams still reluctant to stray from traditional systems that provide more procedural flexibility and safety margins in uncertain intraoperative situations, particularly in centers with high procedural volume and established CPB protocols. Second, cost-effectiveness is still an issue. Although MECC circuits may lessen postoperative complications, they frequently call for specialized tools and training, which not all institutions can afford. Third, major guidelines and surgical societies have not fully endorsed it due to the lack of consistent protocols and solid long-term outcome data, resulting in MECC still playing a specialized role and being typically utilized in centers with specialized knowledge for elective, low-risk, or minimally invasive procedures. Standardization of training and protocols, wider clinical validation, and technological simplification may be necessary for its eventual incorporation into mainstream practice [103,104,105,107].

### 10.2. Biocompatibility and Modern Circuit Design

At the same time, improvements in biocompatible materials, such as oxygenators lined with phosphorylcholine, tubing coated with heparin, and other surface modifications, have greatly decreased immunological activation, protein adsorption, and blood trauma, lowering the risk of systemic inflammatory response syndrome (SIRS), limit platelet consumption, and maintain coagulation function [114,115,116].

The incorporation of smart technology into contemporary CPB machines is another significant innovation. Perfusionists can precisely regulate perfusion pressure, blood temperature, and oxygen delivery with the use of real-time hemodynamic monitoring tools such as pressure sensors, flow meters, gas analyzers, and automated feedback loops, promoting ongoing quality improvement by supporting data collection and outcome analysis in addition to improving intraoperative safety [117,118].

Lastly, enhancing the CPB experience has been greatly aided by better oxygenators and pump designs. While contemporary centrifugal pumps offer non-occlusive blood propulsion that reduces the risk of hemolysis and embolic complications, membrane oxygenators today offer effective gas exchange with little resistance and less shear stress [119,120,121].

When taken as a whole, these material and technical developments have increased CPB’s suitability for a wider range of surgical settings and patient demographics, with modern CPB systems providing a higher level of safety, control, and physiologic harmony than ever before, especially in neonatal and pediatric surgery, reoperative procedures, and patients with multiple comorbidities. These advances in circuit design and biomaterials have translated into measurable clinical benefits, including lower transfusion requirements, reduced incidence of postoperative systemic inflammatory response, and improved outcomes in high-risk populations such as neonates and patients with severe comorbidities. Longitudinal data suggest that such refinements have been pivotal in decreasing CPB-related morbidity and mortality over the last decades.

Such innovations also carry an ethical imperative. Every advancement in circuit design should not only improve efficiency but demonstrably reduce risks of hemolysis, infection, or inflammatory injury, ensuring that technical progress translates into tangible patient benefit.

### 10.3. Off-Pump Cardiac Surgery

Off-pump cardiac surgery, sometimes referred to as beating heart surgery, emerged in the second half of the 20th century concurrent with the ongoing improvement of CPB systems, avoiding the physiological disturbances linked to extracorporeal circulation by enabling surgeons to perform procedures, and most notably coronary artery bypass grafting (CABG), without the use of CPB [122].

Off-pump techniques are justified by their ability to lessen the systemic inflammatory response, coagulation abnormalities, and neurological complications that are occasionally associated with CPB, using specialized stabilization devices to immobilize a specific region of the beating heart in order to precisely anastomose bypass grafts while the heart continues to pump and circulate blood naturally [123,124]. Off-pump surgery proponents point out a number of possible advantages:

- Decreased neurocognitive deterioration, especially in older patients.
- Reduced prevalence of renal dysfunction and atrial fibrillation following surgery.
- Quicker recovery periods and shorter ICU stays.
- Reduced chance of complications from transfusions.

However, there are drawbacks to the strategy as well, posing a significant technical learning curve involved in operating on a moving heart, and some research indicates that graft patency problems or incomplete revascularization might happen more frequently than with traditional, on-pump CABG, with complex or multi-vessel disease potentially still requiring CPB support, so not all patients are good candidates [125]. Large randomized trials provide a nuanced picture: in ROOBY, off-pump CABG was associated with inferior graft patency and worse composite outcomes, including at 5 years; by contrast, the multinational CORONARY trial found broadly similar major outcomes at 30 days and 1 year, with some perioperative advantages for off-pump but no long-term mortality benefit [126,127]. While the ROOBY trial reported inferior graft patency and outcomes with off-pump CABG, the multinational CORONARY trial demonstrated broadly similar survival but emphasized the importance of surgical expertise. These divergent results underline that outcomes depend as much on operator skill and institutional context as on the technique itself.

The debate over off-pump versus on-pump coronary artery bypass grafting (CABG) has evolved in recent years from a simple binary assessment to a more complex analysis focused on patient selection, surgeon skill, and institutional experience, with current evidence indicating that off-pump CABG offers a clear short-term advantage in lowering the risk of perioperative stroke, though there is still some disagreement. However, it may come at the expense of higher rates of mid-term coronary reintervention and a possible increase in long-term mortality, resulting in off-pump surgery now generally considered a useful supplement to traditional on-pump procedures rather than a better option, being especially helpful for high-risk or fragile patients with numerous comorbidities, where reducing systemic insult is a top concern [125,128,129]. As surgical technology develops further, hybrid approaches that combine robotic support, off-pump techniques, and minimally invasive access are broadening the scope of cardiac surgery, further customizing care, and enhancing results for specific patients.

### 10.4. ECMO and ECLS: Expanding the Role of Mechanical Circulatory and Respiratory Support

Over the past few decades, extracorporeal membrane oxygenation (ECMO), which started out as an experimental adjunct to CPB, has developed into a vital, life-saving technique in contemporary intensive care, with ECMO, which falls under the larger category of extracorporeal life support (ECLS), being currently being used extensively in the treatment of refractory respiratory and cardiac failure, helping to bridge the gap between advanced organ failure and long-term support, recovery, or transplantation [130].

ECMO was initially intended for brief use during surgery or in neonatal care, but it has since grown in scope and technical sophistication to become a standard in critical care for both adults and children. When it was used as a life-saving measure for patients with severe acute respiratory distress syndrome (ARDS) who were not responding to mechanical ventilation during the COVID-19 pandemic, its adaptability and effectiveness were especially highlighted [131,132,133]. ECMO is typically divided into two primary configurations, each of which is designed to meet particular clinical requirements [134]:

- Veno-venous (VV) ECMO: Provides respiratory support only by draining venous blood, oxygenating it via a membrane oxygenator, and returning it to the venous system. It is used primarily in cases of severe pulmonary failure, such as ARDS, pneumonia, or trauma-related lung injury.
- Veno-arterial (VA) ECMO: Supports both circulatory and respiratory function, making it suitable for cardiogenic shock, post-cardiotomy failure, or massive pulmonary embolism. It can temporarily replace the function of both the heart and lungs, offering time for recovery or definitive treatment.

The safety and accessibility of ECMO have been further improved by technological advancements [131,134]:

- Its portability and miniaturization now allow for use in emergency rooms, ambulances, and even during inter-hospital transfers.
- Heparin-coated, closed-loop circuits lower the risk of thrombosis and inflammation.
- Real-time pressure, flow, and gas exchange monitoring is provided by built-in sensors, and increased physiological stability is guaranteed by bubble detectors and integrated heaters.

ECMO is used in many different contexts nowadays [134,135]:

- Neonatal and pediatric intensive care units, where it continues to save infants with congenital diaphragmatic hernia or persistent pulmonary hypertension.
- Cardiothoracic operating rooms, as an adjunct or backup to CPB.
- Emergency departments and trauma centers, where it may be initiated in cases of cardiac arrest (“ECPR”) when conventional resuscitation fails.

ECMO is now widely acknowledged as a versatile bridging platform in critical care, enabling time-sensitive decisions to be made under more stable physiological conditions, in addition to its immediate life-support function, offering short-term support while the heart or lungs recover when a patient has reversible lung injury or fulminant myocarditis [136,137]. In order to preserve organ function and enhance candidacy, bridge-to-transplantation entails keeping patients awaiting heart or lung transplantation at appropriate perfusion and oxygenation levels. When patients require long-term mechanical circulatory support, ECMO can act as a bridge-to-device, keeping them stable until ventricular assist devices (VADs) or complete artificial hearts are implanted. In acute, undifferentiated cases, like severe cardiac arrest or shock of uncertain origin, where ECMO buys time to assess neurologic status, identify underlying pathology, and determine long-term prognosis, the bridge-to-device model is especially crucial. A bridge-to-weaning strategy has also been implemented by certain institutions, which uses ECMO to gradually wean critically ill patients off of continuous ventilator support, highlighting ECMO’s function as an adaptive, strategic tool in the treatment of complex cardiopulmonary failure, in addition to its role as a rescue therapy [136,137,138].

Randomized and registry evidence supports the selective use of ECMO in severe respiratory failure: the CESAR trial showed improved survival without severe disability at 6 months in patients referred to an ECMO center; EOLIA, while underpowered for its primary endpoint, demonstrated a strong trend toward lower 60-day mortality with early VV-ECMO and permitted crossover rescue. Large ELSO registry analyses during the COVID-19 era further report survival around 60% in adult ARDS when ECMO is applied within experienced networks [139,140,141,142]. CESAR and EOLIA illustrate the potential of ECMO to improve survival in severe ARDS, yet also highlight the ongoing controversy surrounding optimal timing and patient selection. Registry analyses during COVID-19 confirm the benefit of ECMO in selected patients but also underscore the resource-intensiveness of this therapy.

The development of ECMO is a reflection of a paradigm shift in medicine’s approach to organ failure and resuscitation, as well as a technological advancement, with its growing use in emergency medicine, critical care, and transplantation being a logical progression of the advancements initially made with CPB. When combined, they create a range of extracorporeal life support techniques that increase survival, broaden the range of available treatments, and push the limits of what is feasible from a surgical and medical standpoint. These technical advances inevitably raise ethical questions of patient selection and resource allocation, particularly in settings where ECMO availability is limited. Balancing lifesaving potential with fairness remains a core challenge.

### 10.5. Beyond Bypass: Minimally Invasive, Hybrid, and Transcatheter Alternatives

Although CPB is still a fundamental component of open heart surgery, minimally invasive, robotically assisted, and transcatheter techniques have dramatically increased over the past 20 years with the goal of minimizing surgical trauma, avoiding CPB completely, or integrating CPB in a more targeted and selective way, becoming part of a larger movement toward patient-specific treatments, quicker recuperation, and better long-term results.

Through tiny thoracic incisions, minimally invasive and robotically assisted cardiac surgery allows surgeons to accomplish intricate procedures, like mitral valve repair, atrial septal defect closure, and even coronary revascularization, often without the need for a sternotomy or complete CPB. MECC or partial bypass circuits are frequently used when extracorporeal support is required, lessening the systemic impact. Particularly in high-volume centers, robotic platforms improve accuracy, lessen operator fatigue, and pave the way for truly micro-access cardiac surgery [143,144]. In cardiac surgery, total percutaneous bypass is the least invasive method of creating extracorporeal circulation and can produce excellent results. Even with large-bore cannulas, the use of contemporary vascular closure devices (VCDs) makes percutaneous access easier and allows for efficient hemostasis after cardiovascular procedures, enhancing the outcomes of groin cannulation, total percutaneous CPB can augment the benefits of robotic surgery [145].

With cutting-edge imaging technologies like intraoperative CT, fluoroscopy, and 3D echocardiography, hybrid operating rooms enable real-time procedural navigation and the fusion of catheter-based and surgical procedures. For example, off-pump left internal mammary artery (LIMA) grafting and percutaneous coronary interventions (PCI) may be combined in hybrid coronary revascularization to balance minimal invasiveness and durability [146,147].

Transcatheter therapies, which increasingly take the place of or postpone conventional surgical procedures, are equally revolutionary. Transcatheter aortic valve replacement (TAVR), which is currently frequently utilized in patients who are not good candidates for open heart surgery, is the most prominent example [148,149], with other innovations including percutaneous closure of septal defects [150], endovascular aneurysm repair (EVAR/TEVAR) [151], and transcatheter mitral valve repair/replacement (TMVR) [149], providing efficient substitutes with shorter hospital stays and fewer complications because they usually do not require CPB in patients who are carefully chosen.

The emergence of hybrid and transcatheter solutions indicates a growing commitment to less invasive, patient-tailored approaches, even though CPB is still necessary for many complex procedures, frequently supplementing CPB rather than replacing it with perfusion technologies modified for shorter runs, partial support, or as backup systems in high-risk interventions [152].

The field of cardiac surgery is becoming more and more defined by its capacity to combine the best open, minimally invasive, and endovascular techniques, all supported by decades of advancements in extracorporeal circulation, rather than by a single technique or philosophy as these tools continue to develop.

## 11. Translational Impact and Future Directions in Cardiopulmonary Support

The creation of CPB, which turns theoretical anatomy and physiology into life-saving clinical practice, is a victory of biomedical innovation. However, the history of extracorporeal support does not stop with the heart–lung machine; rather, it keeps developing in tandem with advancements in precision medicine, systems biology, artificial intelligence, and materials science, with its future depending on radically rethinking how circulation and oxygenation can be supported, enhanced, and even replaced, in addition to making surgery safer and more successful [153].

The incorporation of artificial intelligence (AI) into perfusion management is among the most exciting advancements. In order to provide predictive modeling of oxygen delivery, hemodynamic stability, and coagulation risk, machine learning algorithms are being trained on enormous datasets of intraoperative parameters, helping perfusionists make decisions in real time, enhancing patient safety by supporting automated control loops that react dynamically to physiologic changes during surgery [154,155,156,157,158,159].

Early translational studies suggest feasibility of ML-based decision support to anticipate perfusionist actions and optimize flow/oxygen-delivery targets in real time, potentially reducing operator variability and standardizing outcomes across centers [159,160]. AI-assisted systems may outperform traditional perfusion monitoring by predicting oxygen delivery thresholds and adjusting pump flow in real time, though these findings require validation in larger clinical trials. The incorporation of AI into CPB management has changed the way things work, especially for perfusionists who now have access to tools that enable clinical decision-making that goes well beyond conventional monitoring. With the ability to process enormous volumes of physiological data in real time and provide predictive, adaptive recommendations that optimize perfusion parameters, AI-based systems are quickly evolving from assistive to collaborative partners in perfusion practice. Goal-directed therapy that adapts dynamically to patient-specific factors like flow, oxygen delivery, hematocrit, and systemic vascular resistance is made possible by these platforms’ integration of machine learning algorithms that have been trained on sizable datasets of past CPB cases, expanding on decades of advancements in goal-directed perfusion strategies, targeting customized thresholds rather than predetermined norms in an effort to preserve homeostasis during CPB [154]. Perfusionists can now move from reactive to proactive management, improving safety and accuracy, thanks to AI systems that can continuously analyze data from pressure transducers, gas exchange monitors, and flow sensors [155,156].

Condello et al. [157] go on to discuss the use of closed-loop systems and management algorithms that serve as early-warning systems by supporting hemodynamic control and warning teams of trends before clinical deterioration happens, being especially helpful in high-risk or complex cases where quick, well-informed intervention is necessary due to rapid perfusion fluctuations. Additionally, AI aids in training, quality enhancement, and data standardization, which lowers inter-operator variability and improves consistency amongst institutions. Even though there are still issues to be resolved, like interoperability, clinical validation, and ethical transparency, the use of AI in CPB management has the potential to completely transform the role of the perfusionist by combining human knowledge with machine accuracy and opening the door to a new era of data-driven, intelligent extracorporeal circulation [157]. Early clinical applications of AI-assisted perfusion are already emerging, with pilot studies demonstrating improved prediction of oxygen delivery thresholds and automated adjustments of pump flow in real time. These systems not only support perfusionists in critical decision-making but also hold promise for standardizing outcomes across institutions by reducing operator-dependent variability. Future integration with big data registries may further enhance predictive modeling and refine patient-specific perfusion strategies.

Preclinical testing of CPB components is being transformed by developments in organ-on-chip technologies, which are miniature platforms that mimic human organ function. Researchers can now test the biocompatibility and performance of novel materials and medications under controlled conditions by simulating the mechanical and biological environment of blood vessels, lungs, and myocardium on microfluidic chips, significantly speeding up the development of safer and more efficient extracorporeal devices [159,160,161,162,163].

Nanotechnology, meanwhile, is creating new avenues for oxygen transport, blood preservation, and inflammatory control. One day, red blood cell-like nanoparticles might function as synthetic oxygen carriers, and other nanoparticles might be anti-inflammatory, lowering the risk of complications related to CPB like organ dysfunction or systemic inflammatory response syndrome (SIRS), with coatings with nanoscale topography also being investigated in order to stop thrombosis and protein fouling in CPB circuits [162,164].

In the future, studies are moving closer to creating a complete artificial heart–lung system, which would be a small, wearable, or implanted device that could sustain cardiopulmonary function completely for prolonged periods of time. Although this technology is still in its conceptual stages, it has the potential to significantly influence the treatment of end-stage lung and heart disease, making it more difficult to distinguish between destination therapy and bridge therapy [165].

The move to customized perfusion techniques is equally revolutionary, with future perfusion protocols being customized to a person’s genetic coagulation profile, inflammatory markers, or metabolic demands in light of the expanding availability of genomic, proteomic, and metabolomic data, maximizing results and reducing risk. A long-standing objective in pharmacotherapy, personalized medicine is now entering the operating room [166].

Even though they are still in their infancy, these developments follow a similar path: the ongoing pursuit of improving extracorporeal support’s accuracy, versatility, and integration with the body’s inherent physiology, with multidisciplinary convergence, bringing together medicine, engineering, data science, and systems biology to expand what is surgically and clinically feasible, being the key to the future of CPB and extracorporeal life support, from AI-assisted perfusion to circuits enhanced by nanotechnology. Alongside technical promise, AI introduces ethical considerations of transparency, accountability, and the necessity of maintaining human oversight in life-sustaining decisions.

## 12. Global Perspectives and Access to CPB Technology

Although CPB and extracorporeal life support (ECLS) systems, like ECMO, have revolutionized critical care and surgery in high-income nations, their accessibility and use are still very uneven worldwide, with the widespread adoption of these life-saving technologies having been severely hampered in low- and middle-income countries (LMICs) by a lack of access to specialized equipment, skilled workers, and long-term support infrastructure [167,168].

Access to ECMO circuits, oxygenators, or perfusion consumables may be irregular or unaffordable, and many cardiac centers in resource-constrained environments continue to use antiquated or refurbished CPB machines, resulting to life-saving treatments such as open heart surgery or ECMO for respiratory failure being either inaccessible or limited to a small population, frequently in urban academic hospitals or private institutions [169,170].

A number of innovations, including portable ECMO devices, simplified MECC platforms, and inexpensive perfusion systems, have been developed to close this gap, being made to be more cost-effective, durable, and simple to use, all of which are essential for deployment in low-resource settings. Concurrently, telemedicine-based perfusion education, international training collaborations, and humanitarian cardiac surgery missions have been crucial in fostering long-term growth and enhancing local capacity [170,171].

These initiatives are not without ethical complexity, though, with sustainability, equity, and resource allocation continuing to be major concerns. In healthcare systems that still lack basic services, should expensive interventions like ECMO be given priority? How can health systems that are experiencing shortages in primary care, vaccines, or necessary medications responsibly incorporate advanced therapies?

In addition to technological innovation, addressing these disparities calls for a coordinated effort to improve global health equity, reform policies, and form partnerships to increase capacity. Making sure that CPB and ECLS technologies are distributed ethically and fairly is still a crucial challenge, and a moral requirement, for the international medical community as they develop. Encouragingly, several initiatives are underway to bridge these disparities, including the development of simplified, low-cost CPB platforms for pediatric surgery in resource-limited settings and international collaborations offering tele-education for perfusionists. Programs such as mobile ECMO teams and portable bypass systems have also begun to extend access to extracorporeal support beyond large tertiary centers, suggesting a future in which lifesaving technologies may become more globally available.

## 13. The Role of Perfusionists and Multidisciplinary Teams

With the introduction of CPB, heart surgery was transformed, and the crucial field of perfusionists, specialists in charge of running and keeping an eye on life-supporting equipment like CPB and ECMO, was born. A highly qualified perfusionist, whose proficiency in controlling extracorporeal circulation is essential to patient survival, is the driving force behind every successful CPB procedure, with his role having changed along with CPB technology, progressing from an early technical assistant to a crucial clinical decision-maker in the operating room team [172,173].

The demands on perfusionists have increased dramatically as extracorporeal technologies have developed and become essential in both surgical and critical care settings, expected to manage intricate physiological parameters, remain vigilant throughout high-stakes procedures, and react quickly to emergencies. The use of ECMO increased during the COVID-19 pandemic, increasing workload and stress, especially during interfacility transports, prolonged treatments, and infection risk exposure. Perfusionists frequently go unappreciated despite their vital contributions, dealing with long hours, emotional exhaustion, and a lack of institutional and public recognition. Supporting the sustainability and expansion of this vital but often disregarded profession requires addressing burnout, enhancing working conditions, and recognizing the vital role of perfusionists [172]. The COVID-19 pandemic highlighted this evolution, with perfusionists increasingly assuming leadership roles in the initiation and management of ECMO across intensive care and emergency settings. As extracorporeal support expands into areas beyond traditional cardiac surgery, their expertise is becoming central to critical care delivery, underscoring the need for structured training, support systems, and professional recognition.

Today’s perfusionists must complete extensive training and certification requirements, which frequently include graduate-level coursework in biomedical engineering, pharmacology, and physiology, their duties going beyond pump operation and circuit setup to include emergency response coordination, anticoagulation management, and real-time hemodynamic optimization [173,174].

Surgeons, anesthesiologists, perfusionists, and nursing staff must work together seamlessly for cardiac surgery to be successful, operating in high-stakes situations where trust, flexibility, and communication are critical. But this intensity has a price: as surgical volumes and technological complexity rise, concerns about burnout, mental exhaustion, and a lack of personnel are becoming more pressing [175,176,177].

Recognizing and supporting the contributions of perfusionists is vital to sustaining excellence in cardiac care. As extracorporeal technologies expand into intensive care, emergency medicine, and transport, the role of these professionals will only become more central—and more deserving of recognition.

## 14. Limitations

This study traces the development of CPB from ancient theory to contemporary clinical practice, providing a comprehensive conceptual and historical overview, also recognizing some limitations, though. The analysis inevitably leaves out some region-specific developments, technical subtleties, and institutional milestones that have also contributed to the field because of its broad chronological and thematic scope, with the rich diversity of contributions from around the world, especially from non-Western medical traditions in the modern era, having not been fully explored, despite efforts to highlight important individuals and innovations. Although the early historical sections are proportionally longer than in many contemporary reviews, this emphasis is intentional, as the manuscript aims to demonstrate how ancient and pre-modern physiological concepts directly shaped the intellectual pathway toward the development of cardiopulmonary bypass.

Furthermore, the manuscript does not perform a systematic review of the literature or offer a thorough quantitative assessment of CPB outcomes, despite integrating technological, clinical, and ethical viewpoints. Rather, the method is primarily interpretive and narrative, with the goal of placing CPB in the larger context of medical innovation and history, with future research potentially gaining more understanding of the lived experience underlying this remarkable medical accomplishment by examining regional disparities in greater detail, comparing CPB protocols, and gathering firsthand reports from patients and clinicians.

Finally, as CPB technologies continue to evolve, their adoption must be continually assessed against ethical frameworks. Issues such as equitable access to advanced circuits and ECMO, the responsible integration of AI into clinical decision-making, and the sustainability of resource-intensive technologies underscore that technical innovation cannot be separated from ethical responsibility.

## 15. Conclusions

One of the most amazing stories in medical history is the evolution of cardiopulmonary bypass, which spans from ancient Greek philosophy to state-of-the-art extracorporeal technologies, with the development of the heart–lung machine, a breakthrough that transformed cardiac surgery and critical care, being the result of centuries of anatomical research and physiological understanding, as this manuscript has examined. We looked at the ethical conundrums of early experimentation, the critical role of surgical teams and perfusionists, and the growth of extracorporeal life support through ECMO in addition to the technical advancements, while also emphasizing inequalities in access around the world and projected future paths influenced by nanotechnology, AI, and personalized medicine. Collectively, these viewpoints demonstrate how CPB is a dynamic and ever-evolving interface between science, innovation, and human life rather than just a technological accomplishment, with its future promising even more integration, accuracy, and worldwide impact, and its legacy continues to inspire.

## Data Availability

The data presented in this study are available upon request from the corresponding author.
